# Pricing Services: An Underutilized Policy Instrument for Access and Quality

**DOI:** 10.1177/11786329221127943

**Published:** 2022-10-19

**Authors:** Sarah L Barber, Naoki Ikegami

**Affiliations:** 1 World Health Organization Centre for Health Development, Kobe, Japan; 2 Kurume University, Japan, and Keio University, Minato-ku, Tokyo, Japan

**Keywords:** Price, regulation, health policy, health economics

## Abstract

We aim to introduce the readers of this special collection to the importance of pricing health and long-term care services, a topic covered in 2 recent joint World Health Organization/Organization for Economic Co-operation and Development (WHO/OECD) publications. The special issue will focus on country experiences in pricing setting and regulation, best practices, and areas for future research.

While payment methods have received a great deal of attention among policymakers and practitioners, less attention has been paid to price setting and how it can also contribute to broader system objectives. However, if prices are set too high or too low, they can easily overshadow the incentives in payment mechanisms. Prices should reflect actual costs and also take into consideration broader health system goals including efficiency toward better health outcomes. If not, unintended negative consequences could arise. In example, if prices are set too low for capitation payments, this could result in low quality care, provider selection of healthier patients, or referral of complex cases that require a higher intensity of services to another service provider. If fee-for-service payments are low, providers may try to compensate by increasing volume and providing additional (unnecessary) services. In this special collection, Kees et al discuss the challenges of cost-based price setting to ensure that prices reflect costs and value.

Controlling the growth of health care spending while maintaining or increasing access is a major policy priority. Generally, health care spending increases at rates higher than general inflation. This is a function of both volumes of care and prices. As illustrated by Lorenzoni and Dougherty,^3^ wide price variation can be seen across countries. Increases in both prices and volumes can be attributed to the adoption and dissemination of new technologies, increases in national income, insurance design, and demographics. In this context, price setting serves as an instrument to reduce or increase volumes of certain services or treatment modalities to control costs. In addition, the volume of specific items could be controlled by setting the conditions of billing the item. For example, in Japan, in cancer, the billing of Positron Emission Tomography (PET) scans is restricted to those who already have a confirmed diagnosis to confirm its spread and may not be used for screening purposes.

As discussed by Tangcharoensathien et al in their paper about balance billing, health care providers are in some cases permitted to bill patients at prices higher than the regulated rates, and the difference is paid by the patient. Under balance billing, services could be underprovided where patients are unable to pay—even though the services are part of the benefits package and valued by communities and societies. In this case, although the government may be commitment to delivering basic services to all citizens, some of the financial burden would be shifted to individuals. However, in long-term care, billing extra for “hotel services” such as room and meal charges would be not always be considered as basic services.

While a great deal of experience has been published in high-income countries, low- and middle-income countries are facing pressures for higher value in health spending. Shankar Prinja et al discuss the experience in India where price setting is a central part of the government’s large-scale health insurance program. One challenge is the relationship and application of prices in the private health care sector. Rising health care spending has pressured policymakers to maximize all available health resources toward meeting these expectations. Governments frequently draw on the private sector to promote sustainability, optimal use of resources, and increased choice of care. Honda et al discuss the challenge of harnessing resources and efficiency gains while addressing the market failures and equity concerns associated with the private financing of health care. Population aging is resulting in greater demand for services appropriate to the needs of older adults. Manuel Flores et al discuss the role of consumers as purchasers of home care services and the challenges and barriers they face in attaining quality care. The extent to which different norms could or should be applied in long-term care must be examined in each country.

Ultimately, however, pricing is not only about covering costs but also providing the right incentives. Pricing, payment systems, their regulatory frameworks, and the conditions of billing can be powerful tools to drive broader health system goals for delivering the appropriate volume of quality services. We hope this supplement serves to draw greater attention and research to pricing as a policy instrument to achieve better value for health spending.
